# MethHaplo: combining allele-specific DNA methylation and SNPs for haplotype region identification

**DOI:** 10.1186/s12859-020-03798-7

**Published:** 2020-10-12

**Authors:** Qiangwei Zhou, Ze Wang, Jing Li, Wing-Kin Sung, Guoliang Li

**Affiliations:** 1 National Key Laboratory of Crop Genetic Improvement, Huazhong Agricultural University, Wuhan, 430070 China; 2 Agricultural Bioinformatics Key Laboratory of Hubei Province, Hubei Engineering Technology Research Center of Agricultural Big Data, 3D Genomics Research Center, College of Informatics, Huazhong Agricultural University, Wuhan, 430070 China; 3 College of Life Science and Technology, Huazhong Agricultural University, Wuhan, 430070 China; 4 Department of Computer Science, National University of Singapore, Singapore, 117417 Singapore; 5 Department of Computational and Systems Biology, Genome Institute of Singapore, Singapore, 138672 Singapore

**Keywords:** DNA methylation, Allele-specific DNA methylation, CTCF, SNP, Allele-specific gene expression

## Abstract

**Background:**

DNA methylation is an important epigenetic modification that plays a critical role in most eukaryotic organisms. Parental alleles in haploid genomes may exhibit different methylation patterns, which can lead to different phenotypes and even different therapeutic and drug responses to diseases. However, to our knowledge, no software is available for the identification of DNA methylation haplotype regions with combined allele-specific DNA methylation, single nucleotide polymorphisms (SNPs) and high-throughput chromosome conformation capture (Hi-C) data.

**Results:**

In this paper, we developed a new method, MethHaplo, that identify DNA methylation haplotype regions with allele-specific DNA methylation and SNPs from whole-genome bisulfite sequencing (WGBS) data. Our results showed that methylation haplotype regions were ten times longer than haplotypes with SNPs only. When we integrate WGBS and Hi-C data, MethHaplo could call even longer haplotypes.

**Conclusions:**

This study illustrates the usefulness of methylation haplotypes. By constructing methylation haplotypes for various cell lines, we provide a clearer picture of the effect of DNA methylation on gene expression, histone modification and three-dimensional chromosome structure at the haplotype level. Our method could benefit the study of parental inheritance-related disease and hybrid vigor in agriculture.

## Background

Genetic variations have vital effects on an organism’s phenotype, which can be studied with haplotypes. Haplotypes can refer to the combinations of alleles or a group of single nucleotide polymorphisms (SNPs) found on the same chromosome [1]. Haplotype analysis has applications in the diagnosis of genetic diseases, ancestry inference, and drug design [2–4]. Generally, the differences in the two haplotypes of an individual’s genome are mainly caused by heterozygous single nucleotide polymorphisms (SNPs) where the haplotypes contain two distinct alleles. In diploid genomes, some parental alleles exhibit different DNA methylation patterns, which may cause variance in individuals with respect to resistance to diseases and responses to therapeutic drugs [5–8]. Therefore, DNA methylation, in addition to SNPs, is vital for distinguishing haplotypes.

In addition, there is a link between SNPs and DNA methylation, and they synergistically regulate gene expression. The association between SNPs and gene expression can be mediated by DNA methylation [9]. In recent years, epigenetics-GWAS (Genome-wide association studies) has been proposed, which can accurately detect the association between DNA methylation, histone modification and phenotype [10]. A series of genes related to diabetes [11, 12], the psychosis of humans [13], and the flowering and development of plants [10] have been analyzed by GWAS and epigenetics-GWAS [14]. These results suggested that there are significant interactions between DNA methylation and SNPs in the regulation of physiological functions [9]. Therefore, appropriate software is needed to obtain haplotype blocks from accurate allele-specific DNA methylation and SNP haplotype information for DNA methylation related study.

DNA methylation has different methylation patterns in alleles, which can lead to allele-specific expressed genes and X chromosome inactivation [5, 6, 15]. However, the differential DNA methylation patterns in alleles remain unclear to date. Thus far, we know that SNPsplit [16] can distinguish allele-specific DNA methylation (ASM) in adjacent regions according to SNP loci, but the results depend heavily on the distribution of SNPs in the whole genome. MONOD2 [17] and MethPipe [18] can perform allele specific DNA methylation analysis, but they didn't combine SNP information and Hi-C interaction information.

In this study, we developed a new method, MethHaplo, for haplotype region identification with ASM and SNPs from whole-genome bisulfite sequencing (WGBS) data. The haplotype identification is carried out by analyzing the ASM patterns of the nearby cytosines using a hypergeometric distribution and an iterative extending approach. The correctness of haplotype identification was validated on human cell lines (K562 and HepG2) and laboratory-generated *Arabidopsis* F1 hybrids with known haplotype information. Our results showed that the haplotype identification could not only reconstruct longer haplotype regions but also link more SNPs to the relative haplotype blocks. The analyses in the A549, GM12878, HepG2, HUES64, IMR90, and K562 human cell lines showed that haplotype identification could reveal some specific patterns in DNA methylation from WGBS, gene expression from RNA sequencing (RNA-Seq), histone modification from chromatin immunoprecipitation sequencing (ChIP-Seq) and three-dimensional chromosome architecture at the haplotype level.

In summary, MethHaplo can help us identify better haplotypes, which may contribute to DNA methylation association analysis, SNP association analysis and the study of parental inheritance-related disease and hybrid vigor in agriculture [19–22].

## Implementation

### MethHaplo: Haplotype region identification with allele-specific DNA methylation, SNPs and Hi-C data

**Fig. 1:**
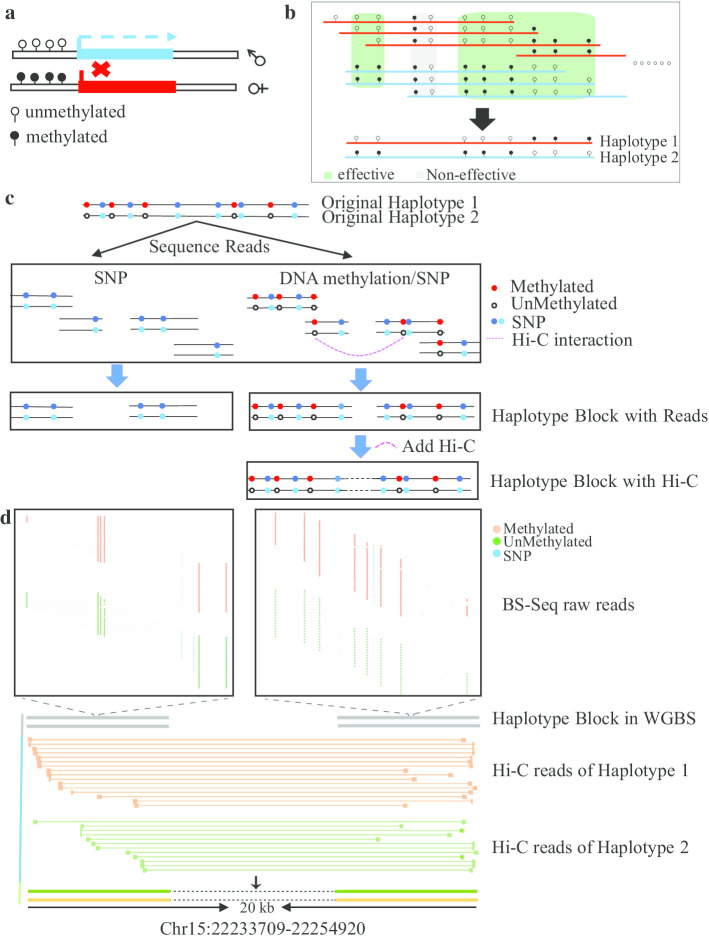
Illustration of haplotype region identification. **a** ASM and its effect on gene expression. Solid black circles represent methylated sites, and open circles represent unmethylated sites. In this example, the gene in the paternal haplotype without DNA methylation is expressed. **b** Schematic of haplotype region identification with ASM. **c** Schematic of haplotype region identification with SNPs, ASM and Hi-C data. The left flow chart represents the haplotype region identification with SNPs only, and the right flow chart represents haplotype region identification with ASM, SNPs, and Hi-C data. **d** A specific example of haplotype region identification with ASM, SNPs and Hi-C

Alleles may have different patterns of DNA methylation, and allele-specific DNA methylation affects the level of allele-specific expression (Fig. 1a) [15]. According to the sequence reads covering the ASM sites, DNA methylation haploid blocks can be assembled. Based on this idea, we proposed a new method, MethHaplo, for haplotype region identification with ASM and SNPs. Figure 1b shows a diagram of haplotype region identification with ASM. In the assembly process, all the totally methylated (methylation level > 0.9) and the totally unmethylated (methylation level < 0.1) sites (highlighted with the gray box in Fig. 1b) were removed first, and only partially methylated cytosine sites, denoted as effective sites, were retained for haplotype region identification.^1^ We used $M_{ri}$ to represent the methylation status on the genome cytosine site $i$ from the read $r$ as methylated, and $U_{ri} { }$ to represent the methylation status on the genome cytosine site $i$ from the read $r$ as unmethylated. Then, we counted the number of reads with different combinations of methylation status in the adjacent sites covered by the same reads:

1 $$N_{ij} = N\left( {M_{ri} ,{ }M_{rj} } \right),{ }N\left( {M_{ri} ,{ }U_{rj} } \right),{ }N\left( {U_{ri} ,{ }M_{rj} } \right),{ }N\left( {U_{ri} ,{ }U_{rj} } \right)$$

where *j* is the genomic cytosine site adjacent to the cytosine site *i* with larger genomic coordinate and sufficient coverage, $N\left( {M_{ri} ,{ }M_{rj} } \right)$ is the number of reads with both methylated status at the adjacent effective cytosine sites *i* and *j*, and others are similar with different combinations of methylation status. The range of *i* and *j* is from 1 to the length of the chromosome considered.

When SNPs were considered, we used $R_{ri}$ to represent the SNP status in the read *r* covered on the genome site $i$ was the same as the reference genome and used $V_{ri}$ to represent that the SNP status in the read *r* covered on the genome site $i$ was different from the reference genome. Similarly, we counted the number of reads with different combinations of methylation status and SNP status in the adjacent sites covered by the same reads:

2 $$N_{ij} = N\left( {M_{ri} ,{ }R_{rj} } \right),{ }N\left( {M_{ri} ,{ }V_{rj} } \right),{ }N\left( {U_{ri} ,{ }R_{rj} } \right),{ }N\left( {U_{ri} ,{ }V_{rj} } \right)$$

The P value between the adjacent effective cytosine sites is calculated by the hypergeometric distribution [23]. The formula is as follows:

3 $$p_{ij} = hyper\_g\left( {N\left( {M_{ri} ,{ }M_{rj} } \right),{ }N\left( {{*},{ }M_{rj} } \right),{ }N\left( {{*},{ }U_{rj} } \right),{ }N\left( {M_{rj} ,{ *}} \right)} \right)$$

For each paired adjacent DNA methylation sites, Fisher's exact test was performed if each valid cytosine site was covered by at least n bisulfite sequencing reads (e.g., n = 4). The P values were adjusted with the false discovery rate (FDR) method for multiple hypothesis testing, proposed by Benjamini and Hochberg [24]. If the association between two adjacent sites meets the programmed criteria, these two adjacent sites were defined as the ASM sites and assigned to a haplotype block. Then, the association between the site with larger coordinate and its next adjacent site was calculated. On this basis, the block was further extended until the final haplotype result was obtained.

A region is defined as an allele-specific DNA methylation region (ASMR) if the region meets the following criteria: (1) the adjusted P value of the adjacent sites is smaller than the predefined threshold (default: 0.05); (2) The sum of the maximum value and the second maximum value of the combination of the adjacent sites exceeds 90% of the total number of covered reads; and (3) The ratio of the maximum value of the combination of the adjacent sites to the second largest value is less than 2. Here the maximum value and the second maximum value are from the values in the formula (1) if only DNA methylation is considered, and are from the values in the formula (2) if DNA methylation and SNPs are considered.

High-throughput chromosome conformation capture (Hi-C) is a method that can generate reads with spatial proximity but with a long genomic distance [25]. Hi-C data have been proposed for assisting genome assembly by linking the scaffolds [26]. To further improve the haplotype region identification, we combined Hi-C data in the method and linked haplotype blocks with longer genomic distance by Hi-C interaction reads to obtain longer blocks. In our tool, we developed the HapScore algorithm to combine the WGBS data and Hi-C data to obtain longer haplotypes (Fig. 1c). A specific example of haplotype region identification with ASM, SNPs and Hi-C is shown in Fig. 1d.

For Hi-C-based haplotype region identification, the heterozygous SNP sites were used for haplotype region identification. Because DNA methylation has different methylation patterns in the positive and negative strands, we distinguished BAM files according to the positive and negative strands with SAMtools. Then, we used the HapCUT2 algorithm [27] to complete the haplotype region identification with the effective DNA methylation information and the heterozygous SNP information. The haplotype region identification diagram is shown in Fig. 1c.

We developed the HapScore algorithm to merge the WGBS data haplotype with the Hi-C data haplotype. We defined $B_{i}$ as the haplotype results of the WGBS data at the i-th position on the genome and $H_{i}$ as the haplotype results of the Hi-C data at the i-th position on the genome. We set $S_{i}$ as the score obtained by the $B_{i}$ and $H_{i}$.

4 $$S_{i} = \left\{ {\begin{array}{*{20}l} {k; } \hfill & { if\;B_{i} = = H_{i} } \hfill \\ { - k; } \hfill & { if\;B_{i} = = revH_{i} } \hfill \\ {0;} \hfill & { others } \hfill \\ \end{array} } \right.$$

Then, in a certain overlapping haplotype interval between the two sets of data, the consistency score (HapScore) can be calculated as:

5 $$H_{s} = \left| {\mathop \sum \limits_{l = 1}^{n} S_{l} } \right|$$

where *n* represents the number of SNPs in the overlapping block.

When the HapScore is greater than the threshold defined in the program, the merge is completed according to the two sets of haplotype results, and a new haplotype block result is produced.

In the haplotype length analysis portion, we used the HapCUT2 algorithm and HapScore algorithm. The ASM analysis used the hypergeometric algorithm. The format of the ASM result file is as follows: "chromosome start end LengthofBlock NumberofCytosines".

### Allele-specific gene expression analysis

Raw RNA-Seq reads were first trimmed using FastQC (https://www.bioinformatics.babraham.ac.uk/projects/fastqc/) and Trimmomatic [28] with default parameters to remove the adaptors and the low-quality reads. Clean reads were mapped to the human reference genome hg38 using Hisat2 [16], and then SAMtools [29] was used to sort the BAM file. Allele-specific expression genes (ASEGs) were detected by ASEQ [30].

### Allele-specific CTCF analysis

The low-quality read trimming and the artificial sequence trimming were performed with FastQC and Trimmomatic. The genome was masked (a genome in which all known SNP positions were masked with the ambiguity base ‘N’) before alignment by the genome mask script in SNPsplit [16] with default parameters. Clean reads were mapped to the hg38 masked genome using Bowtie2 [31], and then SAMtools was used to sort the BAM file. The peaks were processed with MACS2 [32]. The sorted BAM file was then processed with SNPsplit. The allele-specific CTCF binding peaks must satisfy "total number of allele reads in the peaks are larger than 10" and “the fold change between alleles is larger than 2”. The percentage of the allele-specific CTCF peak was calculated by the number of allele-specific CTCF peaks divided by the total number of CTCF peaks.

### Allele-specific Hi-C interaction analysis

First, we masked all bases in the genome that were genotyped as SNPs in either the mouse genome or human genome by the genome mask script in SNPsplit. These bases were masked as "N" to reduce reference bias mapping artifacts. The raw reads were aligned to the hg38 (human) masked genome or mm10 (mouse) masked genome with HiC-Pro [33], and then SAMtools was used to sort the BAM file. The sorted alignment BAM file was processed with SNPsplit. Then, we removed all the unsigned alignment reads, which could not be distinguished from parental alleles. The allele-specific Hi-C interaction bins must satisfy "the total number of reads in the bins is larger than 20" and “the fold change between alleles is larger than 2”.

### Methy-HiC analysis

Raw reads were first trimmed as paired-end reads using Trimmomatic with the default parameters to remove the adaptors and the low-quality reads. We aligned Methy-HiC reads to the mouse reference genome mm10 using Burrows-Wheeler Aligner (BWA) and Bhmem (https://bitbucket.org/dnaase/bisulfitehic/src/master/). The DNA methylation ratio was calculated by BatMeth2-calmeth [34], and then SAMtools was used to convert the reads to the BAM format. MethHaplo was used for haplotype region identification (with HapCUT2 algorithms [27]).

## Results

### MethHaplo yields longer haplotypes

**Fig. 2:**
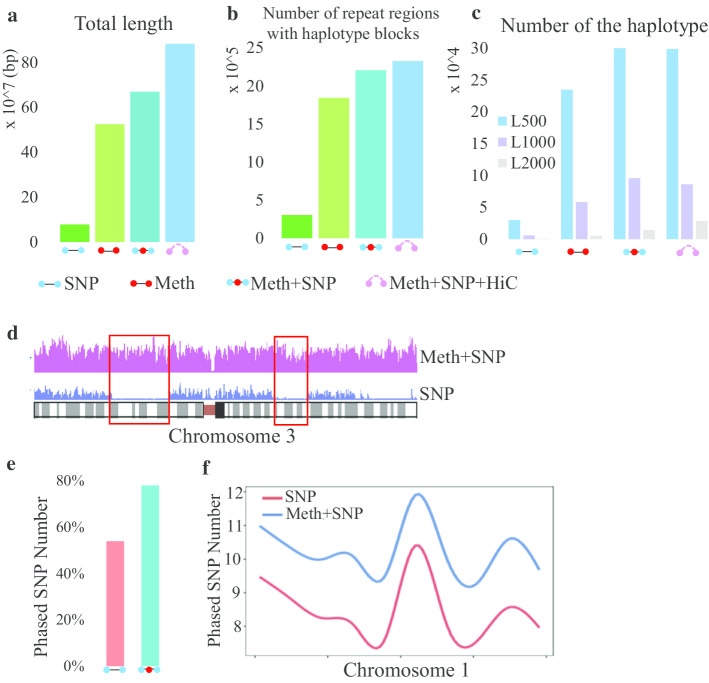
Properties of haplotype regions identified in the A549 cell line. **a** Total lengths of haplotype blocks from different conditions for haplotype region identification. **b** Repeat regions covered by haplotype blocks from different conditions for haplotype region identification. **c** The distribution of haplotype block lengths. For each condition, the haplotype blocks are grouped by (1) block length > 2 kb, (2) 1–2 kb, and (3) 500 bp–1 kb. **d** The coverage of the haplotype blocks on the genome. Pink is for the haplotype results with SNPs and ASM combined, and blue is for haplotype results with SNPs only. **e** The percentage of phased SNPs in the haplotype results. The total number of phased SNPs was 866, 434 (percentage of phased variants: 866, 434/1, 616, 824 = 54%) in the condition of haplotype region identification with SNPs only. When SNPs and ASM were combined for haplotype region identification, the total number of phased SNPs was 1, 261, 123 (1, 261, 123 /1, 616, 824 = 78%). **f** The distribution of phased SNPs on chromosome 3 under two conditions (with or without ASM)

To investigate the performance of MethHaplo, we performed haplotype region identification under 4 different conditions: (1) haplotype regions identified with SNP information only, (2) haplotype regions identified with ASM only, (3) haplotype regions identified with ASM and SNPs, and (4) haplotype regions identified with ASM, SNPs and Hi-C data. The data we used were the publicly available whole-genome bisulfite DNA methylation sequence data and Hi-C data from different human cell lines (A549, GM12878, HepG2, HUES64, IMR90, and K562). Using the A549 cell line as an example, the results in Fig. 2a show that the total length of the haplotype identified with ASM and SNP information was seven times longer than that with SNP information only. The total length of haplotype blocks identified with ASM only was also much longer than that assembled with SNPs only.

Figure 2b shows that there are more haplotype blocks with a length of 2000 bp or more assembled using ASM and SNPs than those assembled using SNPs only, and the haplotype region identification was further improved with Hi-C information. We counted the number of repeats covered by haplotype blocks. Figure 2c shows that there were more repeats covered by haplotype blocks from ASM and SNPs. Finally, we analyzed the distribution of haplotype region identification blocks on chromosomes. The coverage of haplotype blocks identified with both ASM and SNPs was much higher than that assembled with SNPs only (Fig. 2d). The haplotype regions identified from HepG2, K562, and IMR90 cell lines are shown in Additional file 1: Figure S1.

To determine the effects of ASM on SNP assembly, we calculated the proportions of SNPs assigned to different haplotype blocks with and without ASM information. Figure 2e, f show that more (~ 24%) heterozygous SNPs could be assembled in the haplotype blocks by ASM and SNPs than those with SNP information only.

### Verification of the accuracy of MethHaplo in different scenarios

**Fig. 3:**
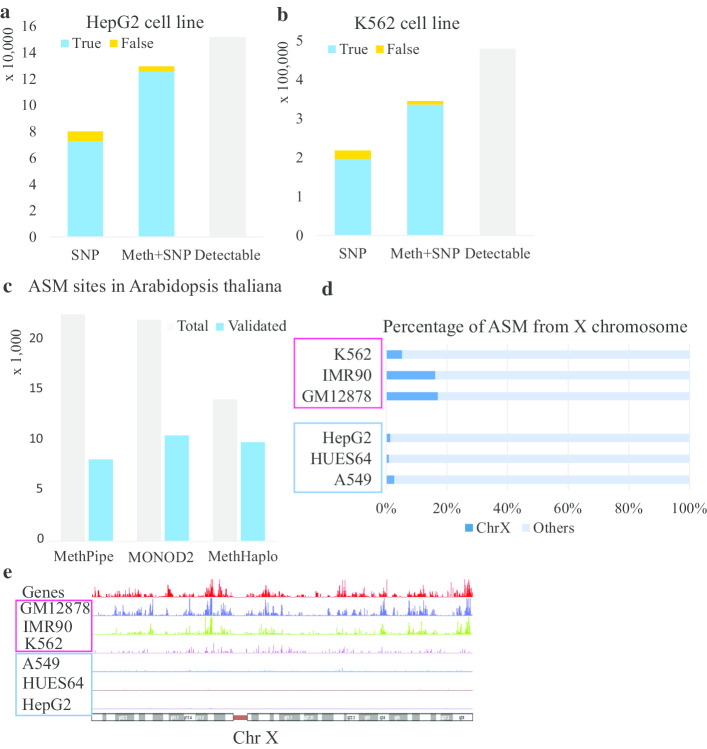
Evaluation of the accuracy of MethHaplo in different approaches. **a**, **b** Validation of the haplotype results compared with the latest published HepG2 (**a**) and K562 (**b**) cell line haplotype genomes. **c** Validation of the ASM results in *Arabidopsis thaliana* F1 hybrid data. **d** The proportion of ASM on chromosome X versus other chromosomes in male cell lines (blue box) and female cell lines (red box). **e** The densities of ASM on chromosome X for the male cell lines and the female cell lines

To assess the correctness of our identified haplotype regions, we used the latest published haplotype genomes for the K562 and HepG2 cell lines [35, 36] as references to validate the haplotype regions identified by MethHaplo. Figure 3a, b show that MethHaplo could generate more correct SNPs and fewer incorrect SNPs in haplotypes than those from SNP information alone.

To further verify the accuracy of MethHaplo, we analyzed the characteristics of ASM. At present, the tools that can detect ASM without relying on SNP information include MONOD2 [17], MethPipe [18] and Amrfinder [19]. In fact, Amrfinder and MethPipe are the same software programs used in the detection of ASM. To provide an accurate criterion for measuring ASM accuracy, we grew the *Arabidopsis thaliana* strains Cvi and Ler and their hybrid and obtained the WGBS data of the Cvi, Ler and F1 hybrids. MethPipe, MONOD2 and MethHaplo were used to detect ASM in the F1 hybrid, and the results were further compared with the parents (Cvi and Ler). MethHaplo and MONOD2 have higher sensitivity than MethPipe, and the results detected by MethHaplo are more precise (Fig. 3c). The assembly result in F1 hybrids is consistent with the above conclusion that ASM improves the result of haplotype region identification (Additional file 1: Figure S2).

In addition, we analyzed the relationship of ASM with different properties. As reported, ASM is highly correlated with imprinted genes and allele-specific expressed genes [15, 37]. Therefore, we downloaded all known validated human imprinted genes from the "imprinted gene database" (https://www.geneimprint.com/) and analyzed the overlap between ASM genes (ASMGs) and imprinted genes. As expected, imprinted genes significantly overlapped with ASMGs in the tested cell lines (Table 1, P value was calculated by Fisher's exact test). Furthermore, we analyzed the transcriptome data of the tested cell lines, and obtained allele-specific expressed genes (ASEGs). Similarly, ASEGs were significantly enriched with ASMGs (Table 2).

**Table 1:** Overlap between ASMGs and known imprinted genes

Cell line	ASMG	Overlap^a^	P value
A549	1851	19	< 2.2e−16
HUES64	952	25	< 2.2e−16
GM12878	5919	41	< 2.2e−16
IMR90	2556	30	< 2.2e−16
HepG2	1929	26	< 2.2e−16
K562	1758	15	< 2.2e−16

^a^There are 87 known imprinted genes in human genome. For each tested cell line, the second column shows the number of ASMGs and the third column shows the number of overlapped genes between the ASMGs and known imprinted genes

**Table 2:** Overlap between ASMGs and ASEGs

Cell line	ASMG	ASEG	Overlap^a^	P value
A549	1851	1648	141	< 2.2e−16
HUES64	952	342	42	< 2.2e−16
GM12878	5919	1352	316	< 2.2e−16
IMR90	2556	1426	162	< 2.2e−16
HepG2	1929	3175	237	< 2.2e−16
K562	1758	4092	319	< 2.2e−16

^a^For each tested cell line, the second column shows the number of ASMGs, the third column shows the number of ASEGs, and the fourth column shows the number of overlapped genes between the ASMGs and ASEGs

ASM is widely distributed on female X chromosomes for X chromosome inactivation [38]. To test this hypothesis, we analyzed the distribution of ASM regions on all chromosomes. Figure 3d, e show that the proportion of ASM regions on the X chromosome is 6–17% in female cell lines (K562, IMR90, and GM12878). However, the distribution of ASM regions only accounts for 1.4–2.6% on the X chromosome in male cell lines (HepG2, A549, and HUES64). Due to X chromosome inactivation in female cells, there should be more ASM in female cells. Therefore, these results indicate that MethHaplo has very high accuracy.

### Genomic properties of ASM on the whole genome

**Fig. 4:**
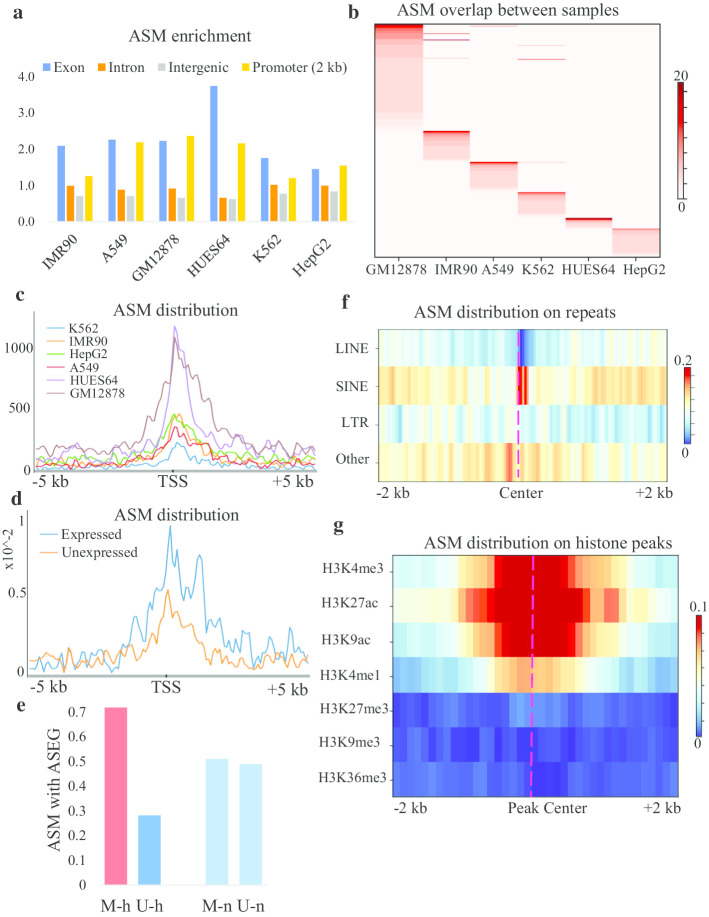
Characterization of ASM around genes, repeats and histone modification peaks. **a** The distribution of ASM in different genomic regions. The X-axis shows the different cell lines. The Y-axis represents the calculated values obtained by dividing the percentage of ASM on the different elements by the percentage of the element length. **b** The overlap of ASM between different cell lines. The genome is partitioned into 2 kb-length bins; then, the number of ASM events is counted for each bin. The heat map shows the counts in all bins for different cell lines. **c** The distribution of ASM across the transcription start site (TSS). **d** The distribution of ASM across TSSs in expressed and unexpressed genes. We divided the genes into expressed and unexpressed genes. The figure shows the distribution of ASM on expressed genes (blue) and unexpressed genes (orange) (using the A549 cell line as an example). **e** The association between the ASM and allele genes. M-h and U-h indicate that the haplotype with high expression is methylated or unmethylated, respectively. M-n and U-n represent whether the haplotype without allele-specific expression is methylated or unmethylated, respectively. **f** The distribution of ASM in repeat regions. The red dashed line represents the center of the repeat regions. **g** The distribution of ASM across different histone modification peaks. The red dashed line represents the center of the peak regions

To characterize the ASM predicted by MethHaplo, we examined the properties of ASM with different genomic properties. We first studied the distribution of ASM in the genome. The results in Fig. 4a show that ASM was highly enriched in the exon and promoter regions. Then, we analyzed the distribution of ASM among all the tested cell lines and found that most ASM was specific to individual cell lines (Fig. 4b, Additional file 1: Table S1). A previous report showed that partial DNA methylation domains could be used to distinguish different cell lines [39]. Here, the high specificity of ASM indicates that ASM can also be used as a marker to distinguish different cell lines.

In imprinting and X chromosome inactivation, ASM leads to monoallelic expression of genes [40, 41]. Thus far, genome-wide studies on the relationship between ASM and allele expression are rare. Thus, we further analyzed the profile of ASM on genes. The results showed that ASM was significantly enriched in the vicinity of the transcription start site (TSS) regions in all tested cell lines (Fig. 4c, Additional file 1: Figure S3). Additionally, combined with the gene expression data, we found that ASM tended to be distributed on expressed genes (Fig. 4d). To understand whether DNA methylation is enriched in highly expressed alleles, we analyzed the association between ASM and allele-specific expression. As shown in Fig. 4e, haplotypes with methylated alleles inside the gene body had higher allele-specific expression. These results demonstrate that the genes in the alleles with gene body methylated are more likely to be expressed. This finding is in accordance with previous studies showing that DNA methylation in the gene body positively regulates gene expression [34, 42].

Finally, we analyzed the distribution of ASM on repeats and histone modification regions. The distribution of ASM in long interspersed repetitive elements (LINEs) is significantly lower than that in other repeat regions (Fig. 4f). ASM distributes more on active histone modification factors (Fig. 4g). These results suggest that ASM is significantly associated with gene expression or gene transcription regulation.

### CTCF tends to distribute on unmethylated haplotypes

**Fig. 5:**
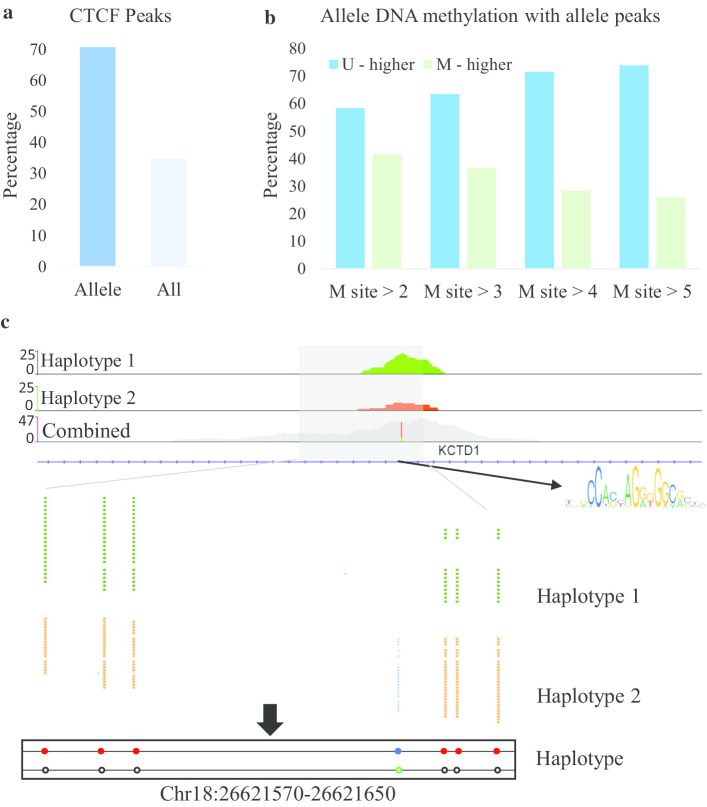
The association between ASM and allele-specific CTCF peaks (ASCPs). **a** The distribution of ASM on the ASCPs. The left and right bars indicate the proportions of ASM in ASCPs and all CTCF peaks, respectively. Fisher's exact test was used in significance analysis. **b** The relationship between ASM and ASCPs. U-higher and M-higher means higher CTCF haplotypes were unmethylated and methylated, respectively. The M site indicates the number of adjacent methylation sites in the ASM region. **c** An example of ASCPs in the region chr18:26, 621, 440-26, 621, 650. The figure shows all bisulfite reads and the haplotype results in this region

CTCF is one of the most critical regulatory factors and plays a vital role in the spatial architecture of chromosomes and gene expression [43–45]. Studies have reported that CTCF binding sites are sensitive to DNA methylation [46, 47]. Here, we aim to determine whether a similar relationship exists between DNA methylation and CTCF at the haplotype level. MethHaplo can help in this analysis. First, we analyzed the distribution of ASM on the allele-specific CTCF (AS-CTCF) binding sites and found that ASM was highly concentrated in the AS-CTCF binding regions (Fig. 5a). Furthermore, we calculated the impact of DNA methylation on CTCF binding at the haplotype level. The alleles tend to be unmethylated when the haplotype has higher CTCF binding (Fig. 5b). An example of the relationship between ASM and AS-CTCF shows that the unmethylated haplotype has a higher CTCF peak (Fig. 5c). In short, CTCF tends to be distributed on unmethylated haplotypes. This result is consistent with the reported conclusion that CTCF is sensitive to DNA methylation [46, 47].

### High association between spatially adjacent ASM sites in haploid three-dimensional structure

**Fig. 6:**
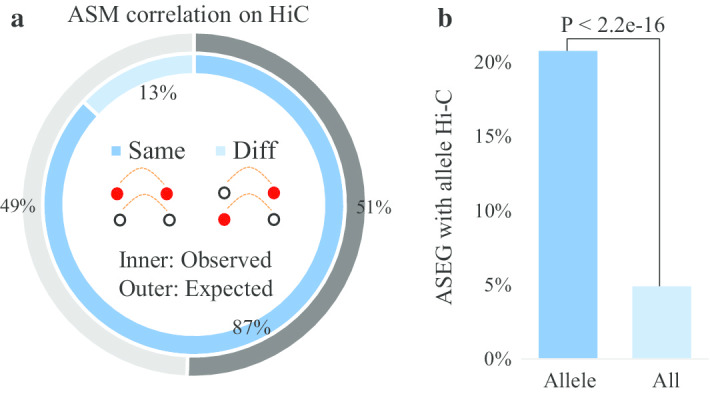
The haplotype region identification and analysis combined with Hi-C data. **a** The distribution of DNA methylation on Hi-C reads at the haplotype level. The inner circle represents the proportion of Hi-C reads with the same or different DNA methylation status at the two ends of individual paired-end reads. The outer circle represents the proportion of reads with the same or different DNA methylation status at the two ends of individual paired-end reads when the pairing of the paired-end Hi-C reads was randomly shuffled (P value < 2.2e−16, Fisher's exact test). **b** The relationship between ASEGs and Allele-specific HiC bins (ASHs). The left and right bars represent the proportions of ASEG in ASHs and all-HiC bins, respectively. Fisher's exact test was used in significance analysis

In our method, the Hi-C reads with SNP information were used to link different haplotype blocks. To understand the association between adjacent ASMs in spatial structure, we obtained all the phased Hi-C reads covered by hetero-SNPs. We calculated the association between ASM at both ends of haplotype-HiC interaction reads. Eighty-seven percent of the ASM sites showed the same methylation pattern on both sides of haplotype-HiC interaction reads (Fig. 6a). These results indicate that ASM also has a high association between spatially adjacent regions of haploid three-dimensional structure.

Moreover, we explored the relationship between alleles and the three-dimensional structure of haploid genomes. The results showed that ASEGs were considerably enriched in the AS-HiC region (Fig. 6b).

Thus, by combining Hi-C interaction information, longer haplotypes can be assembled with MethHaplo, which can benefit many analyses at the haplotype scale, such as ASM and ASEG.

### More allele-specific haplotype reads can be obtained from the data with simultaneous detection of DNA methylation and Hi-C

**Fig. 7:**
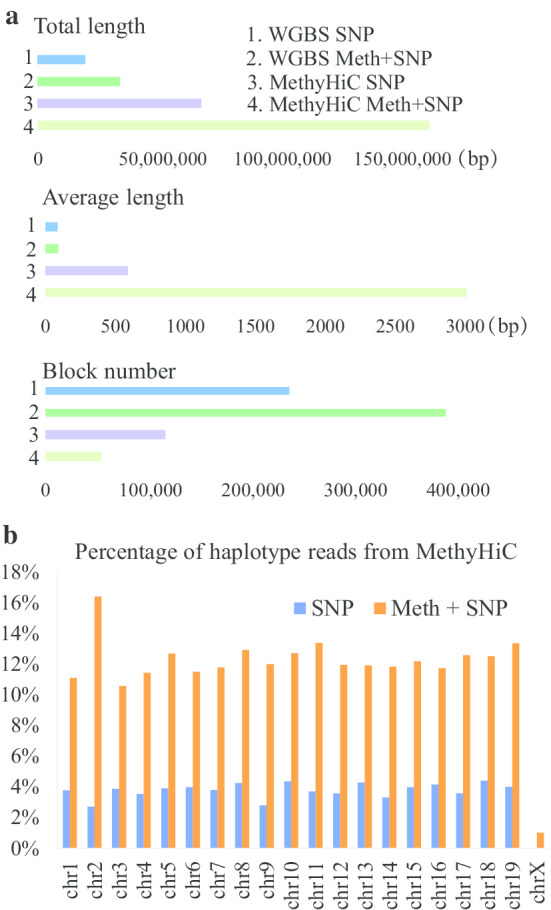
Haplotype region identification with Methy-HiC data. **a** The results of haplotype regions identified from Methy-HiC data, compared with haplotype regions identified from WGBS data. The total length, the average length and the total number of haplotype blocks in chromosome 1 are shown. **b** More allele-specific haplotype reads can be obtained by combining DNA methylation and chromosome conformation information

Methyl-HiC [48] is an experimental technique for the simultaneous detection of DNA methylation and Hi-C that was recently published. To explore whether the Methy-HiC data will lead to longer haplotype results, we used data from the mouse hybrid embryonic stem cell line F123 Methy-HiC [48] and F123 WGBS [49] to complete the haplotype region identification with and without DNA methylation information. The total haplotype length of the Methy-HiC assembly was five times longer than that of the WGBS assembly under the same conditions, and the number of haplotype blocks was obviously lower than that of blocks in the WGBS haplotype region identification results (Fig. 7a). These data indicate that the simultaneous combination of DNA methylation and Hi-C can significantly improve the haplotype region identification results.

Although SNPs can be used to distinguish haplotype alignment reads, the distinguishable reads are very limited. Only 2% of allele-specific interaction reads can be distinguished by SNPs. When DNA methylation information was used to distinguish interaction reads within haplotypes, 10% of allele-specific interaction reads could be distinguished (Fig. 7b, Additional file 1: Figure S4). Therefore, DNA methylation information has important roles in distinguishing the interaction sequences within the haplotype.

## Discussion

In this paper, we proposed a new method, MethHaplo, for haplotype region identification with ASM and SNPs. ASM links more SNP sites in the haplotype region identification, and haplotypes from combined ASM and SNPs are much longer than those from SNPs only. Our results show that the application of ASM could assemble 24% more heterozygous SNPs into different haplotypes (Fig. 2e, f). Moreover, the three-dimensional chromosome structure data (Hi-C) can further enhance the haplotype region identification (Fig. 2a, b). Compared with the recently published haplotype regions of the K562 and HepG2 cell lines identified by whole-genome sequencing, MethHaplo can generate more accurate haplotype regions (Fig. 3a, b).

GWAS is a method to search for variation sequences in human, animal or plant genomes. Epigenetics-GWAS can accurately detect the association between DNA methylation, SNP and phenotype. There is a link between SNP and DNA methylation, and they can synergistically regulate gene expression [9]. However, there is no suitable method to explore the relationship and distribution between SNP and DNA methylation in haplotype block view. In this study, we completed haplotype region identification by combining allele-specific DNA methylation and SNP information. As a result, the association between SNPs and ASM is included in the haplotype region identification blocks, which is critical for the study of DNA methylation and SNP coregulation of gene expression and phenotypic analysis. It will be beneficial to the study of parental inheritance-related disease and hybrid vigor in agriculture.

According to the haplotype region identification results, we can obtain ASM regions. By analyzing the distribution of ASM, we found that ASM sites are concentrated in exonic regions (Fig. 4a). Moreover, ASM is significantly enriched in the TSS regions and distributed in the gene bodies of highly expressed genes (Fig. 4c). In addition, the distribution of ASM among different cell lines is highly specific (Fig. 4b). Thus, ASM can also be used as a marker to distinguish different cell lines. Further analysis of the relationship between ASM and histone modification marks shows that ASM is mainly distributed on the activation-related histone modification marks (Fig. 4g). The specific distribution of ASM on different cells and the significant enrichment of ASM in highly expressed genes indicate that ASM plays an important role in regulating gene expression. Genes on homologous chromosomes have different DNA methylation patterns, and this may have an effect on an individual's resistance to disease and lead to the differences in response to therapeutic drugs [50]. Therefore, accurate analysis of ASM has an essential role in the further exploration and classification of diseases such as cancer. Moreover, the haplotype region identification approach in this work will help to further elucidate DNA methylation on development and disease at the haplotype level.

CTCF is a transcription factor, which performs important functions in the genome, including regulating gene expression and chromatin structure. Related studies have shown that CTCF mutation will lead to changes in three-dimensional structure and gene expression, and high-frequency CTCF mutations have been found in some high-risk tumors [51–53]. Recent studies show that CTCF is enriched in the boundaries of topologically associated domains (TADs) [54], and could be an important protein mediating the long-range chromatin interactions [55, 56]. The results of ASM and AS-CTCF analyses demonstrate that ASM is highly enriched in the AS-CTCF regions, and there is a negative association between them (Fig. 5a). Then, with the Hi-C data analysis, we found that ASM has a very high association between spatially adjacent DNA sequences. Our statistical results also indicate that ASEG is significantly enriched in the AS-HiC region, and a significant positive association exists between ASM and ASEG (Fig. 6a, b). Therefore, we can speculate that the different spatial structures of chromatids are more convenient for ASM to regulate the expression of allele genes. These results provide a basis for further study on the relationship between DNA methylation, gene expression, CTCF and chromatin three-dimensional structure at the haplotype level.

Finally, there could be some limitations in our method. For our design, we expect that MethHaplo can obtain accurate allele specific DNA methylation regions. Still, we cannot exclude the possibility that the heterogeneity of cells can contribute to certain incorrect allele specific DNA methylation regions identified from a population of cells. For example, the DNA methylation profiles from different cell types can be different. If the WGBS data is from such a population of heterogeneous cells, the detected ASM results probably contain differential DNA methylation regions between different cell types as allele-specific DNA methylation, especially when there is no SNP information available. Combining SNP information, it can help to distinguish ASM regions from true haplotype blocks. In our tested data, more than 50% of the ASM regions contain SNPs. Nevertheless, there could be SNPs as somatic mutations in the cells. Such SNPs could not help to accurately distinguish allele specific DNA methylation regions and intercellular differential methylation regions. Currently, the single cell sequencing technology is developing very fast, which can differentiate the genetic and epigenetic information between different cells. We hope that the correct identification of haplotype blocks can be better solved with the single-cell sequencing technology in the future.

## Conclusions

Here, we describe a new method, MethHaplo, for DNA methylation haplotype region identification. We show that by combining ASM and SNPs, MethHaplo obtains haplotype regions that are ten times longer than those with SNPs only. Additionally, MethHaplo can integrate WGBS and Hi-C to further improve the performance of haplotype region identification. As MethHaplo provides an accurate and less fragmented set of haplotypes, new analyses, such as the association between SNPs and DNA methylation, can be carried out at the haplotype level.

## Availability and requirements

- Project name: MethHaplo.
- Project home page: https://github.com/ZhouQiangwei/MethHaplo.
- Operating systems: Linux.
- Programming Languages: C++, Python.
- Other requirements: GCC, SAMtools.
- License: General Public License GPL 3.0.
- Any restrictions to use by non-academics: License required.

## Supplementary information

**Additional file 1.** This additional file contains partial implementation, Figures S1-S5 and Table S1.

## Data Availability

The *Arabidopsis* DNA methylation data generated in this study have been deposited in the Sequence Read Archive (SRA) with accession codes SRR9077100, SRR9077101 and SRR9077102. Human WGBS data used in this study are from the Encyclopedia of DNA Elements (ENCODE) under accession codes ENCFF304DGQ, ENCFF211RZY, ENCSR765JPC and ENCSR890UQO [57], from Gene Expression Omnibus (GEO) with accession numbers GSM1112841 [58] and GSE48592 [59], and the SRA accession code SRX323155 [58]. Hi-C data used are from the ENCODE under accession codes ENCSR662QKG, ENCLB022KPF [57] and ENCSR046XXF [60], and from GEO with the accession numbers GSM1055800 [61] and GSM1551618 [60, 62]. RNA-Seq data used in this study are from ENCODE under accession codes ENCSR937WIG, ENCSR000CPE and ENCSR000CPH [57], and from GEO with accession numbers GSM1112837 [58], GSM2308414 [57], and GSM981249 [63]. ChIP-Seq data used in this study are from the SRA under accession codes SRR357477, SRR5093129 [57], SRR2987869 [64], SRR5093030 [57], SRX190027 [65], SRR357508, SRR577450 [65], SRR2987870 [64], and SRR5093143 [57]. The Methy-HiC sequencing data used in this study are from GEO under the accession code GSE119171 [48]. All data and scripts used in this study are detailed in https://github.com/ZhouQiangwei/MethHaploScripts.

## Abbreviations

ASM: Allele-specific DNA methylation
ASMG: Allele-specific methylation gene
ASEG: Allele-specific gene expression
AS-CTCF: Allele-specific CTCF
AS-HiC: Allele-specific Hi-C interaction
SNP: Single nucleotide polymorphism
WGBS: Whole-genome bisulfite sequencing

## Footnotes

1 The methylation levels (0.1 and 0.9) were empirically defined; Besides, in the software, their values can be adjusted according to the user's preferences.

## References

[1] Altshuler D, Donnelly P (2005). The International HapMap C: a haplotype map of the human genome. Nature.

[2] Clark AG, Weiss KM, Nickerson DA, Taylor SL, Buchanan A, Stengard J, Salomaa V, Vartiainen E, Perola M, Boerwinkle E (1998). Haplotype structure and population genetic inferences from nucleotide-sequence variation in human lipoprotein lipase. Am J Hum Genet.

[3] Wendel B, Flachmeier C, Church GM, Köpke K, Kidd KK, Rohde K, Hoehe MR, Berrettini WH (2000). Sequence variability and candidate gene analysis in complex disease: association of µ opioid receptor gene variation with substance dependence. Hum Mol Genet.

[4] Schwartz R, Clark AG, Istrail S, Guigo R, Gusfield D (2002). Methods for inferring block-wise ancestral history from haploid sequences. Algorithms in bioinformatics: 2002.

[5] Kelly TK, De Carvalho DD, Jones PA (2010). Epigenetic modifications as therapeutic targets. Nat Biotechnol.

[6] Chiba H, Kakuta Y, Kinouchi Y, Kawai Y, Watanabe K, Nagao M, Naito T, Onodera M, Moroi R, Kuroha M (2018). Allele-specific DNA methylation of disease susceptibility genes in Japanese patients with inflammatory bowel disease. PLoS ONE.

[7] Stern JL, Paucek RD, Huang FW, Ghandi M, Nwumeh R, Costello JC, Cech TR (2017). Allele-specific DNA methylation and its interplay with repressive histone marks at promoter-mutant TERT genes. Cell Rep.

[8] Do C, Lang CF, Lin J, Darbary H, Krupska I, Gaba A, Petukhova L, Vonsattel J-P, Gallagher MP, Goland RS (2016). Mechanisms and disease associations of haplotype-dependent allele-specific DNA methylation. Am J Hum Genet.

[9] Wang F, Zhang S, Wen Y, Wei Y, Yan H, Liu H, Su J, Zhang Y, Che J (2014). Revealing the architecture of genetic and epigenetic regulation: a maximum likelihood model. Brief Bioinform.

[10] Cortijo S, Wardenaar R, Colome-Tatche M, Gilly A, Etcheverry M, Labadie K, Caillieux E, Hospital F, Aury JM, Wincker P (2014). Mapping the epigenetic basis of complex traits. Science.

[11] Fuchsberger C, Flannick J, Teslovich TM, Mahajan A, Agarwala V, Gaulton KJ, Ma C, Fontanillas P, Moutsianas L, McCarthy DJ (2016). The genetic architecture of type 2 diabetes. Nature.

[12] Mahajan A, Go MJ, Zhang W, Below JE, Replication DIG, Meta-analysis C, Asian Genetic Epidemiology Network Type 2 Diabetes C, South Asian Type 2 Diabetes C, Mexican American Type 2 Diabetes C, Type 2 Diabetes Genetic Exploration by Nex-generation sequencing in muylti-Ethnic Samples C (2014). Genome-wide trans-ancestry meta-analysis provides insight into the genetic architecture of type 2 diabetes susceptibility. Nat Genet.

[13] Marshall CR, Howrigan DP, Merico D, Thiruvahindrapuram B, Wu W, Greer DS, Antaki D, Shetty A, Holmans PA, Pinto D (2017). Contribution of copy number variants to schizophrenia from a genome-wide study of 41,321 subjects. Nat Genet.

[14] Visscher PM, Wray NR, Zhang Q, Sklar P, McCarthy MI, Brown MA, Yang J (2017). 10 years of GWAS discovery: biology, function, and translation. Am J Hum Genet.

[15] Tycko B (2010). Allele-specific DNA methylation: beyond imprinting. Hum Mol Genet.

[16] Krueger F, Andrews SR (2016). SNPsplit: allele-specific splitting of alignments between genomes with known SNP genotypes. F1000Res.

[17] Guo S, Diep D, Plongthongkum N, Fung H-L, Zhang K, Zhang K (2017). Identification of methylation haplotype blocks aids in deconvolution of heterogeneous tissue samples and tumor tissue-of-origin mapping from plasma DNA. Nat Genet.

[18] Song Q, Decato B, Hong EE, Zhou M, Fang F, Qu J, Garvin T, Kessler M, Zhou J, Smith AD (2013). A reference methylome database and analysis pipeline to facilitate integrative and comparative epigenomics. PLoS ONE.

[19] Fang F, Hodges E, Molaro A, Dean M, Hannon GJ, Smith AD (2012). Genomic landscape of human allele-specific DNA methylation. Proc Natl Acad Sci U S A.

[20] Martos SN, Li T, Ramos RB, Lou D, Dai H, Xu J-C, Gao G, Gao Y, Wang Q, An C (2017). Two approaches reveal a new paradigm of ‘switchable or genetics-influenced allele-specific DNA methylation’ with potential in human disease. Cell Discov.

[21] Lauss K, Wardenaar R, Oka R, van Hulten MHA, Guryev V, Keurentjes JJB, Stam M, Johannes F (2018). Parental DNA methylation states are associated with heterosis in epigenetic hybrids. Plant Physiol.

[22] Kawanabe T, Ishikura S, Miyaji N, Sasaki T, Wu LM, Itabashi E, Takada S, Shimizu M, Takasaki-Yasuda T, Osabe K (2016). Role of DNA methylation in hybrid vigor in Arabidopsis thaliana. Proc Natl Acad Sci U S A.

[23] Johnson NL, Kemp AW, Kotz S (1992). Univariate discrete distributions.

[24] Benjamini Y, Hochberg Y (1995). Controlling the false discovery rate—a practical and powerful approach to multiple testing. J R Stat Soc Ser B Methodol.

[25] Lieberman-Aiden E, van Berkum NL, Williams L, Imakaev M, Ragoczy T, Telling A, Amit I, Lajoie BR, Sabo PJ, Dorschner MO (2009). Comprehensive mapping of long-range interactions reveals folding principles of the human genome. Science (New York, NY).

[26] Burton JN, Adey A, Patwardhan RP, Qiu R, Kitzman JO, Shendure J (2013). Chromosome-scale scaffolding of de novo genome assemblies based on chromatin interactions. Nat Biotechnol.

[27] Edge P, Bafna V, Bansal V (2017). HapCUT2: robust and accurate haplotype assembly for diverse sequencing technologies. Genome Res.

[28] Bolger AM, Lohse M, Usadel B (2014). Trimmomatic: a flexible trimmer for Illumina sequence data. Bioinformatics.

[29] Li H, Handsaker B, Wysoker A, Fennell T, Ruan J, Homer N, Marth G, Abecasis G, Durbin R (2009). Genome project data processing S: the sequence alignment/map format and SAMtools. Bioinformatics.

[30] Romanel A, Lago S, Prandi D, Sboner A, Demichelis F (2015). ASEQ: fast allele-specific studies from next-generation sequencing data. BMC Med Genom.

[31] Langmead B, Salzberg SL (2012). Fast gapped-read alignment with Bowtie 2. Nat Methods.

[32] Zhang Y, Liu T, Meyer CA, Eeckhoute J, Johnson DS, Bernstein BE, Nusbaum C, Myers RM, Brown M, Li W (2008). Model-based analysis of ChIP-Seq (MACS). Genome Biol.

[33] Servant N, Varoquaux N, Lajoie BR, Viara E, Chen C-J, Vert J-P, Heard E, Dekker J, Barillot E (2015). HiC-Pro: an optimized and flexible pipeline for Hi-C data processing. Genome Biol.

[34] Zhou Q, Lim J-Q, Sung W-K, Li G (2019). An integrated package for bisulfite DNA methylation data analysis with Indel-sensitive mapping. BMC Bioinform.

[35] Zhou B, Ho SS, Greer SU, Zhu X, Bell JM, Arthur JG, Spies N, Zhang X, Byeon S, Pattni R (2019). Comprehensive, integrated, and phased whole-genome analysis of the primary ENCODE cell line K562. Genome Res.

[36] Zhou B, Ho SS, Greer SU, Spies N, Bell JM, Zhang X, Zhu X, Arthur JG, Byeon S, Pattni R (2019). Haplotype-resolved and integrated genome analysis of the cancer cell line HepG2. Nucleic Acids Res.

[37] Hamada H, Okae H, Toh H, Chiba H, Hiura H, Shirane K, Sato T, Suyama M, Yaegashi N, Sasaki H (2016). Allele-specific methylome and transcriptome analysis reveals widespread imprinting in the human placenta. Am J Hum Genet.

[38] Zhang Y, Rohde C, Reinhardt R, Voelcker-Rehage C, Jeltsch A (2009). Non-imprinted allele-specific DNA methylation on human autosomes. Genome Biol.

[39] Salhab A, Nordstrom K, Gasparoni G, Kattler K, Ebert P, Ramirez F, Arrigoni L, Muller F, Polansky JK, Cadenas C (2018). A comprehensive analysis of 195 DNA methylomes reveals shared and cell-specific features of partially methylated domains. Genome Biol.

[40] Kerkel K, Spadola A, Yuan E, Kosek J, Jiang L, Hod E, Li K, Murty VV, Schupf N, Vilain E (2008). Genomic surveys by methylation-sensitive SNP analysis identify sequence-dependent allele-specific DNA methylation. Nat Genet.

[41] Chan H-W, Kurago ZB, Stewart CA, Wilson MJ, Martin MP, Mace BE, Carrington M, Trowsdale J, Lutz CT (2003). DNA methylation maintains allele-specific KIRGene expression in human natural killer cells. J Exp Med.

[42] Laurent L, Wong E, Li G, Huynh T, Tsirigos A, Ong CT, Low HM, Kin Sung KW, Rigoutsos I, Loring J (2010). Dynamic changes in the human methylome during differentiation. Genome Res.

[43] Holwerda SJB, de Laat W (2013). CTCF: the protein, the binding partners, the binding sites and their chromatin loops. Philos Trans R Soc Lond Ser B Biol Sci.

[44] Kim S, Yu N-K, Kaang B-K (2015). CTCF as a multifunctional protein in genome regulation and gene expression. Exp Mol Med.

[45] Zuin J, Dixon JR, van der Reijden MIJA, Ye Z, Kolovos P, Brouwer RWW, van de Corput MPC, van de Werken HJG, Knoch TA, van IJcken WFJ (2014). Cohesin and CTCF differentially affect chromatin architecture and gene expression in human cells. Proc Natl Acad Sci.

[46] Renaud S, Loukinov D, Abdullaev Z, Guilleret I, Bosman FT, Lobanenkov V, Benhattar J (2007). Dual role of DNA methylation inside and outside of CTCF-binding regions in the transcriptional regulation of the telomerase hTERT gene. Nucleic Acids Res.

[47] Wang H, Maurano MT, Qu H, Varley KE, Gertz J, Pauli F, Lee K, Canfield T, Weaver M, Sandstrom R (2012). Widespread plasticity in CTCF occupancy linked to DNA methylation. Genome Res.

[48] Li G, Liu Y, Zhang Y, Kubo N, Yu M, Fang R, Kellis M, Ren B (2019). Joint profiling of DNA methylation and chromatin architecture in single cells. Nat Methods.

[49] Xie W, Barr CL, Kim A, Yue F, Lee AY, Eubanks J, Dempster EL, Ren B (2012). Base-resolution analyses of sequence and parent-of-origin dependent DNA methylation in the mouse genome. Cell.

[50] Lachance J (2010). Disease-associated alleles in genome-wide association studies are enriched for derived low frequency alleles relative to HapMap and neutral expectations. BMC Med Genom.

[51] Katainen R, Dave K, Pitkänen E, Palin K, Kivioja T, Välimäki N, Gylfe AE, Ristolainen H, Hänninen UA, Cajuso T (2015). CTCF/cohesin-binding sites are frequently mutated in cancer. Nat Genet.

[52] Umer HM, Cavalli M, Dabrowski MJ, Diamanti K, Kruczyk M, Pan G, Komorowski J, Wadelius C (2016). A significant regulatory mutation burden at a high-affinity position of the CTCF motif in gastrointestinal cancers. Hum Mutat.

[53] Ong C-T, Corces VG (2014). CTCF: an architectural protein bridging genome topology and function. Nat Rev Genet.

[54] Dixon JR, Selvaraj S, Yue F, Kim A, Li Y, Shen Y, Hu M, Liu JS, Ren B (2012). Topological domains in mammalian genomes identified by analysis of chromatin interactions. Nature.

[55] Handoko L, Xu H, Li G, Ngan CY, Chew E, Schnapp M, Lee CWH, Ye C, Ping JLH, Mulawadi F (2011). CTCF-mediated functional chromatin interactome in pluripotent cells. Nat Genet.

[56] Tang Z, Luo OJ, Li X, Zheng M, Zhu JJ, Szalaj P, Trzaskoma P, Magalska A, Wlodarczyk J, Ruszczycki B (2015). CTCF-mediated human 3D genome architecture reveals chromatin topology for transcription. Cell.

[57] Consortium EP (2012). An integrated encyclopedia of DNA elements in the human genome. Nature.

[58] Bernstein BE, Stamatoyannopoulos JA, Costello JF, Ren B, Milosavljevic A, Meissner A, Kellis M, Marra MA, Beaudet AL, Ecker JR (2010). The NIH Roadmap Epigenomics Mapping Consortium. Nat Biotechnol.

[59] Selvaraj S, Dixon RJ, Bansal V, Ren B (2013). Whole-genome haplotype reconstruction using proximity-ligation and shotgun sequencing. Nat Biotechnol.

[60] Rao SSP, Huntley MH, Durand NC, Stamenova EK, Bochkov ID, Robinson JT, Sanborn AL, Machol I, Omer AD, Lander ES (2014). A 3D map of the human genome at kilobase resolution reveals principles of chromatin looping. Cell.

[61] Jin F, Li Y, Dixon JR, Selvaraj S, Ye Z, Lee AY, Yen C-A, Schmitt AD, Espinoza CA, Ren B (2013). A high-resolution map of the three-dimensional chromatin interactome in human cells. Nature.

[62] Sanborn AL, Rao SSP, Huang S-C, Durand NC, Huntley MH, Jewett AI, Bochkov ID, Chinnappan D, Cutkosky A, Li J (2015). Chromatin extrusion explains key features of loop and domain formation in wild-type and engineered genomes. Proc Natl Acad Sci U S A.

[63] Djebali S, Davis CA, Merkel A, Dobin A, Lassmann T, Mortazavi A, Tanzer A, Lagarde J, Lin W, Schlesinger F (2012). Landscape of transcription in human cells. Nature.

[64] Ashoor H, Louis-Brennetot C, Janoueix-Lerosey I, Bajic VB, Boeva V (2017). HMCan-diff: a method to detect changes in histone modifications in cells with different genetic characteristics. Nucleic Acids Res.

[65] Thurman RE, Rynes E, Humbert R, Vierstra J, Maurano MT, Haugen E, Sheffield NC, Stergachis AB, Wang H, Vernot B (2012). The accessible chromatin landscape of the human genome. Nature.

