# Supplementary material for: MethHaplo: combining allele-specific DNA methylation and SNPs for haplotype region identification
Source: BMC Bioinformatics. 2020 Oct 12;21:451. doi: 10.1186/s12859-020-03798-7 (PMC7552496; doi:10.1186/s12859-020-03798-7)
Supplement: Supplementary file 1 — Additional file 1. This additional file contains partial implementation, Figures S1-S5 and Table S1. [file 12859_2020_3798_MOESM1_ESM.pdf]

Additional file to

MethHaplo: Combining Allele-specific DNA Methylation and SNPs for Haplotype Region Identification

Qiangwei Zhou[1,2], Ze Wang[3], Jing Li[3], Wing-Kin Sung[2,4,5], Guoliang Li[1,2*]

Implementation

Whole-genome bisulfite sequencing (WGBS) data analysis

Low-quality read trimming and artificial sequence trimming were performed with FastQC (http://www.bioinformatics.babraham.ac.uk/projects/fastqc/) and Trimmomatic [1]. Clean reads were mapped to the reference genomes hg38 (human) and mm10 (mouse) from UCSC (http://genome.ucsc.edu), or Arabidopsis (TAIR10) from TAIR (https://www.arabidopsis.org) using BatMeth2-align [2]. DNA methylation calling was performed with BatMeth2-calmeth, and the SAM file was converted to the BAM format with SAMtools [3]. SNP calling was performed with BiS-SNP [4].

Arabidopsis WGBS library construction and sequencing

Seeds of A. thaliana were sterilized and sown onto Murashige and Skoog (MS) medium plates containing 1% (wt/vol) sucrose and 0.2% (wt/vol) phytagel. Hybrids between A. thaliana Cape Verde Islands (Cvi-0) and Landsberg erecta (Ler-0) were generated by hand pollination. After 4 d at 4 °C, the plates were transferred to a growth chamber at 22 °C under white light (100 mmol m22 s21) conditions (16 h light/8 h dark). The 10-d-old seedlings were used for WGBS assays, and approximately 200 mg seedlings were pooled in each sample for genomic DNA extraction as described previously [5]. Libraries for high-throughput bisulfite sequencing were generated as described previously [6].

Statistics analysis

In this research, we used Fisher's exact test to complete the significant analysis, and set the model of Fisher's exact test as two-sided.

Performance measures

The methylation status on a cytosine in the parental data from the Arabidopsis thaliana strains Cvi and Ler is treated as the real methylation status. If the methylation status of a cytosine is different in Cvi and Ler, this site is considered as a real allele-specific DNA methylation (ASM) site.

Let TP be the number of true positives (cytosine sites defined as real ASM sites by the parental data, and detected as ASM sites by a tool).

Let FP be the number of false positives (cytosine sites not defined as real ASM sites by the parental data, but detected as ASM sites by a tool).

Let FN be the number of false negatives (cytosine sites defined as ASM sites by the parental data, but not detected in F1 by a tool). Then

Sensitivity, also called 'recall', is defined as $\frac{TP}{TP+FN}$.

Precision is defined as $\frac{TP}{TP+FP}$.

MethHaplo: Effective site selection

When the methylation level is greater than or equal to 0.9 or less than or equal to 0.1, the site is defined as fully methylated or unmethylated. These loci play a very small role in allele specific DNA methylation analysis. Taking IMR90 cell line as an example, we used different DNA methylation levels to include cytosines in the analysis and compared their effects on ASM detection. The DNA methylation levels (ML) chosen for testing were as follows: ML[0-1] for choosing all cytosines with DNA methylation levels between 0 and 1, ML[0.1-0.9] for choosing all cytosines with DNA methylation levels between 0.1 and 0.9, also for ML[0.2-0.8], ML[0.3-0.7], and ML[0.4-0.6] with the different methylation levels .

Figure S5 shows that more ASM regions and sites were detected with DNA methylation levels between 0.1 and 0.9. The methylation levels ML[0-1] includes hypomethylation (< 0.1) and hypermethylation (> 0.9) sites, which affected the extension of ASM block interval around the adjacent methylation sites and led to fewer ASM regions and sites. In addition, after filtering the sites with methylation level smaller than 0.1 or larger than 0.9, ASM detection can be finished in a shorter time.

In addition, considering the different needs of users and different data characteristics, we provide the -f parameter in the program to set the threshold for effective DNA methylation sites.

Supplemental Figures

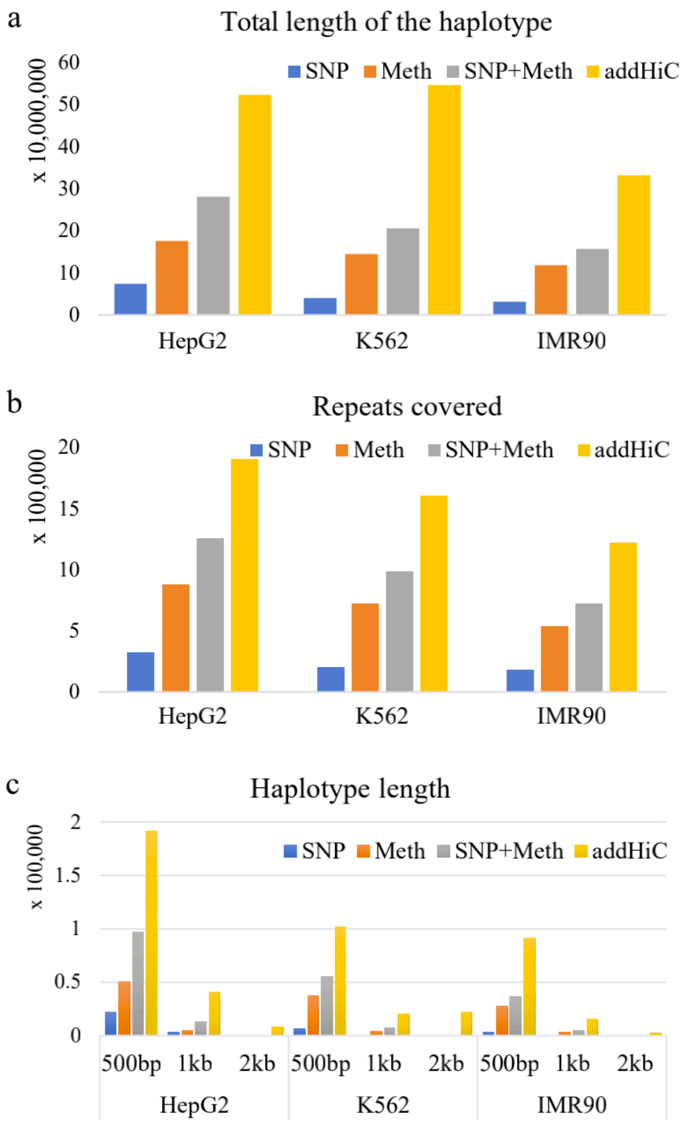

Figure S1. Characteristics of haplotype regions identified in the K562, HepG2 and IMR90 cell lines.

a Total lengths of haplotype blocks from different conditions for haplotype region identification.

b Repeat regions covered by haplotype blocks from different conditions for haplotype region identification.

c The distribution of haplotype block lengths. For each condition, the haplotype blocks are grouped by 1) block length >2 kb, 2) 1 kb–2 kb, and 3) 500 bp–1 kb.

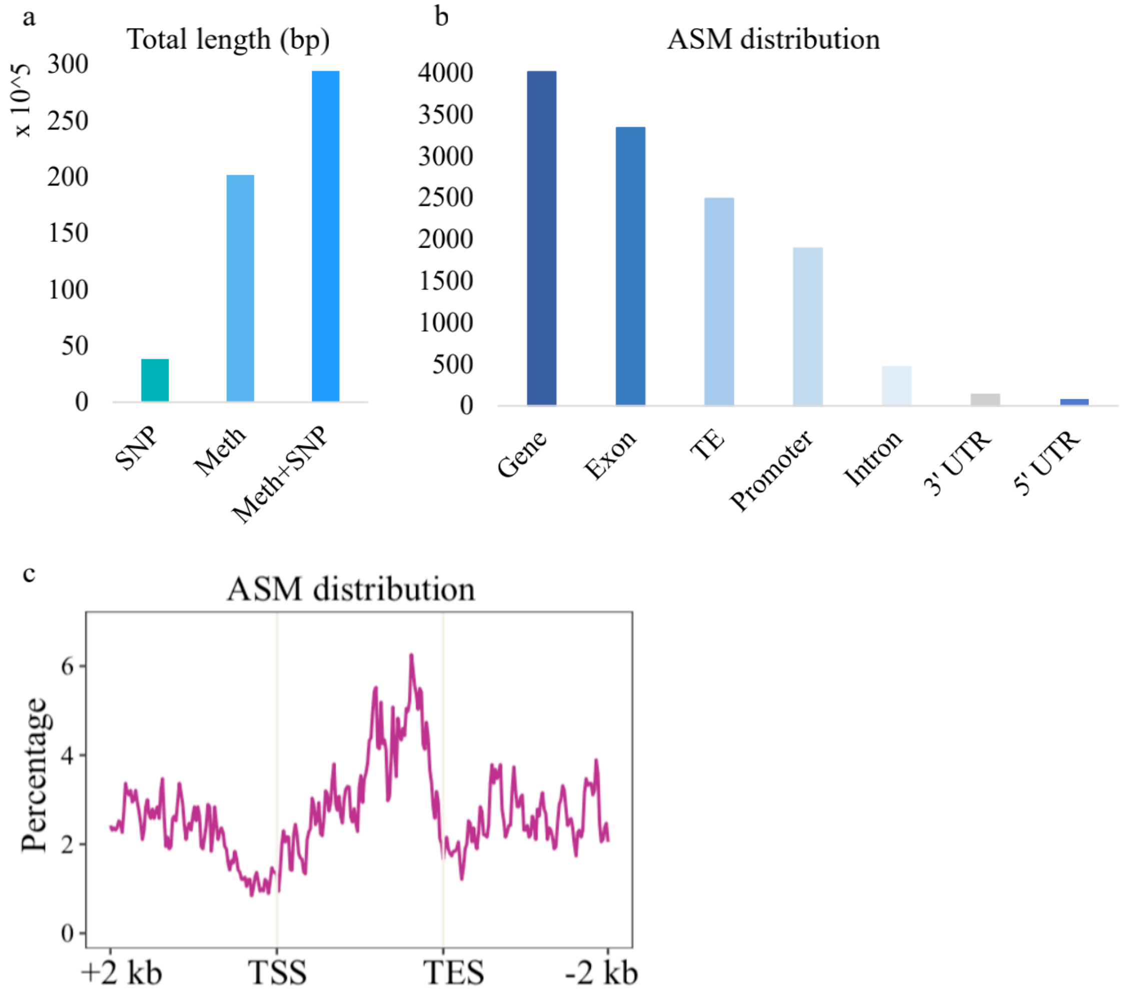

Figure S2. Characteristic of haplotype regions identified in *Arabidopsis thaliana*.

a Total lengths of haplotype blocks from different conditions for haplotype region identification.

b The distribution of ASM in different genomic regions. TE for transposable elements, UTR for un-translated regions.

c The distribution of ASM across the gene bodies. TSS for transcription start sites, TES for transcription end sites.

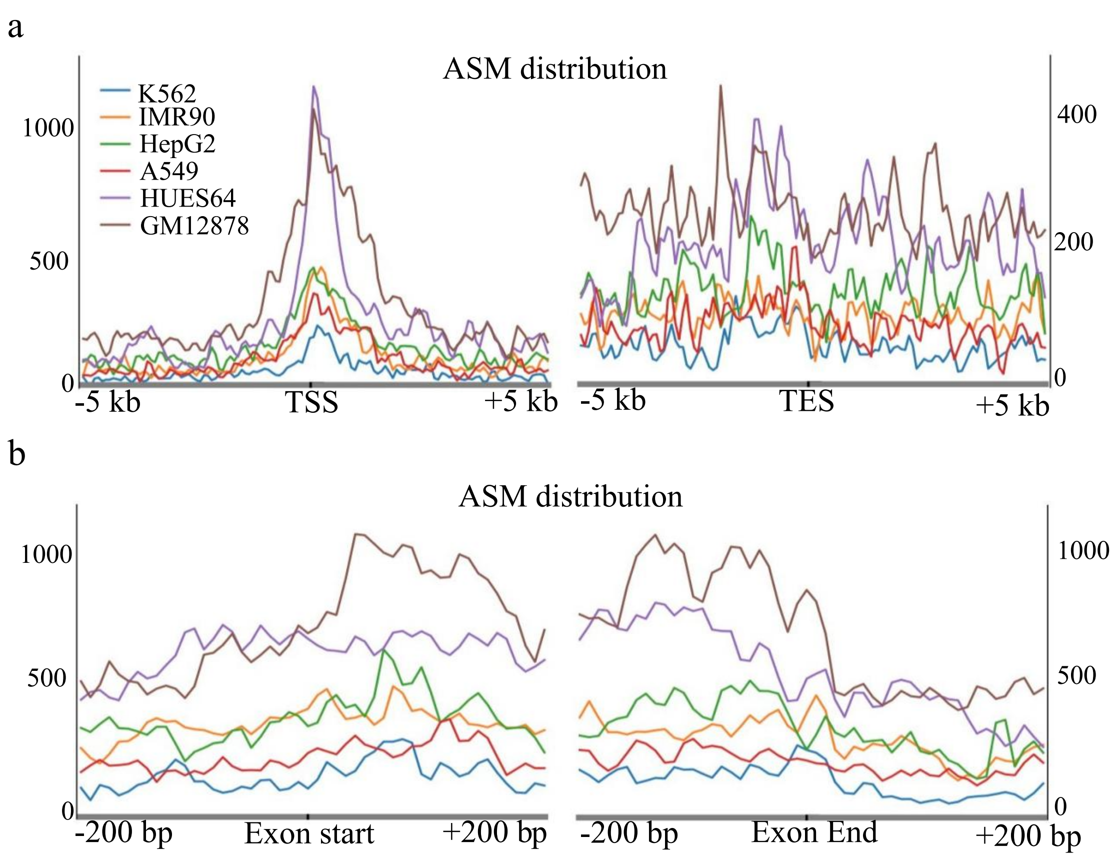

Figure S3. Distribution of ASM regions across gene and exon regions in human cell lines

a Distribution of ASM across gene transcription start sites (TSS) and transcription end sites (TES).

b Distribution of ASM across gene exon start sites and gene exon end sites.

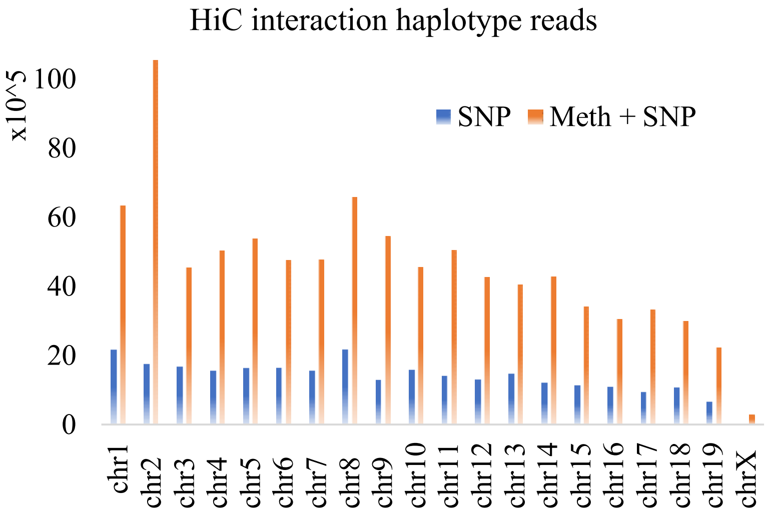

Figure S4. DNA methylation information has important roles in distinguishing the interaction reads within the haplotype.

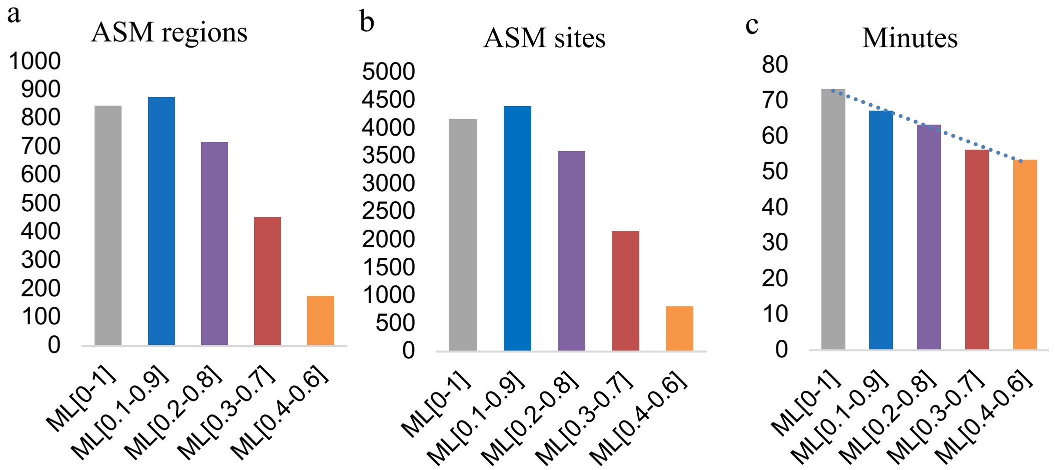

Figure S5. The effect of DNA methylation levels chosen on allele-specific DNA methylation detection.

a ASM regions detected by MethHaplo with different methylation levels.

b ASM sites detected by MethHaplo with different methylation levels.

c Running time (in minutes) from MethHaplo for ASM detecting with different methylation levels.

Table S1. Percentage of overlapped ASMs in different pairs of cell-lines

Overlap	HepG2	HUES64	IMR90	K562	A549
HUES64	3.3%				
IMR90	0.5%	8.8%			
K562	0.1%	0.8%	0.2%		
A549	0.9%	2.5%	1.0%	0.3%	
GM12878	3.4%	6.9%	3.7%	0.9%	2.3%

Reference

1. Bolger AM, Lohse M, Usadel B: **Trimmomatic: a flexible trimmer for Illumina sequence data**. *Bioinformatics* 2014, **30**(15):2114-2120.
2. Zhou Q, Lim J-Q, Sung W-K, Li G: **An integrated package for bisulfite DNA methylation data analysis with Indel-sensitive mapping**. *BMC Bioinformatics* 2019, **20**(1):47.
3. Li H, Handsaker B, Wysoker A, Fennell T, Ruan J, Homer N, Marth G, Abecasis G, Durbin R: **The Sequence Alignment/Map format and SAMtools**. *Bioinformatics* 2009, **25**(16):2078-2079.
4. Liu Y, Siegmund KD, Laird PW, Berman BP: **Bis-SNP: Combined DNA methylation and SNP calling for Bisulfite-seq data**. *Genome Biol* 2012, **13**(7):R61.
5. Allen GC, Flores-Vergara MA, Krasynanski S, Kumar S, Thompson WF: **A modified protocol for rapid DNA isolation from plant tissues using cetyltrimethylammonium bromide**. *Nat Protoc* 2006, **1**(5):2320-2325.
6. Wang Q, Gu L, Adey A, Radlwimmer B, Wang W, Hovestadt V, Bähr M, Wolf S, Shendure J, Eils R *et al*: **Tagmentation-based whole-genome bisulfite sequencing**. *Nat Protoc* 2013, **8**:2022.
